# Altered Expression of Secreted Mediator Genes That Mediate Aggressive Breast Cancer Metastasis to Distant Organs

**DOI:** 10.3390/cancers13112641

**Published:** 2021-05-27

**Authors:** Aparna Maiti, Ichiro Okano, Masanori Oshi, Maiko Okano, Wanqing Tian, Tsutomu Kawaguchi, Eriko Katsuta, Kazuaki Takabe, Li Yan, Santosh K. Patnaik, Nitai C. Hait

**Affiliations:** 1 Department of Surgical Oncology, Roswell Park Comprehensive Cancer Center, Buffalo, NY 14263, USA; Ichiro.Okano@roswellpark.org (I.O.); Masanori.Oshi@roswellpark.org (M.O.); Maiko.Okano@roswellpark.org (M.O.); Tsutomu.Kawaguchi@roswellpark.org (T.K.); Eriko.Katsuta@roswellpark.org (E.K.); kazuaki.takabe@roswellpark.org (K.T.); 2 Department of Molecular & Cellular Biology, Roswell Park Comprehensive Cancer Center, Buffalo, NY 14263, USA; 3 Department of Biostatistics & Bioinformatics, Roswell Park Comprehensive Cancer Center, Buffalo, NY 14263, USA; Wanqing.Tian@RoswellPark.org (W.T.); Yan.Li@Roswellpark.org (L.Y.); 4 Department of Thoracic Surgery, Roswell Park Comprehensive Cancer Center, Buffalo, NY 14263, USA; Santosh.Patnaik@roswellpark.org

**Keywords:** breast cancer, metastases, mediator genes, aggressive subtypes, patients’ survival

## Abstract

**Simple Summary:**

Heterogeneity is the characteristic of breast tumors, making it difficult to understand the molecular mechanism. Alteration of gene expression in the primary tumor versus the metastatic lesion remains challenging for getting any specific targeted therapy. To better understand how gene expression profile changes during metastasis, we compare the primary tumor and distant metastatic tumor gene expression using primary breast tumors compared with its metastatic variant in animal models. Our RNA sequencing data from cells revealed that parental cell and the metastatic variant cell are different in gene expression while gene signature significantly altered during metastasis to distant organs than primary breast tumors. We found that secreted mediators encoding genes (ANGPTL7, MMP3, LCN2, S100A8, and ESM1) are correlated with poor prognosis in the clinical setting as divulged from METABRIC and TCGA-BRCA cohort data analysis.

**Abstract:**

Due to the heterogeneous nature of breast cancer, metastasis organotropism has been poorly understood. This study assessed the specific cancer-related gene expression changes occurring with metastatic breast cancer recurrence to distant organs compared with non-metastatic breast cancer. We found that several secreted mediators encoding genes notably, LCN2 and S100A8 overexpressed at the distant metastatic site spine (LCN2, 5-fold; S100A8, 6-fold) and bone (LCN2, 5-fold; S100A8, 3-fold) vs. primary tumors in the syngeneic implantation/tumor-resection metastasis mouse model. In contrast, the ESM-1 encoding gene is overexpressed in the primary tumors and markedly downregulated at distant metastatic sites. Further digging into TCAGA-BRCA, SCAN-B, and METABRIC cohorts data analysis revealed that LCN2, S100A8, and ESM-1 mediators encoding individual gene expression scores were strongly associated with disease-specific survival (DSS) in the METABRIC cohort (hazard ratio (HR) > 1, *p* < 0.0004). The gene expression scores predicted worse clinically aggressive tumors, such as high Nottingham histological grade and advanced cancer staging. Higher gene expression score of ESM-1 gene was strongly associated with worse overall survival (OS) in the triple-negative breast cancer (TNBC) and hormonal receptor (HR)-positive/HER2-negative subtype in METABRIC cohort, HER2+ subtype in TCGA-BRCA and SCAN-B breast cancer cohorts. Our data suggested that mediators encoding genes with prognostic and predictive values may be clinically useful for breast cancer spine, bone, and lung metastasis, particularly in more aggressive subtypes such as TNBC and HER2+ breast cancer.

## 1. Introduction

Cancer shows organ specificity during metastasis, known as organotropism, an unanswered question in cancer research. Many theories have been proposed to explain the mechanism of metastasis; however, it is still a challenge that is not fully understood. It has been suggested that metastatic dispersion occurs when the primary tumor is very large [1]. Another model postulates that metastatic dissemination occurs in the very early stage of disease progression. Based on all these observations and postulation, it is evident that disseminating cancer cells evolve independent of the primary tumor, that tumor clones can be seeded in parallel or independently to distant sites [2,3]. Thus, it implies that cancer requires systemic treatment at the very stage for efficient elimination [3,4]. Research on metastatic mechanisms is mostly hindering because the lack of a proper experimental model mimics the complex metastatic process. The recent development of patient-derived models and mega-analysis of circulating tumor cells (CTCs) brought insights into metastasis molecular mechanisms and suggested that CTCs take advantage of distant organ supportive niches to establish metastasis. The immune system excludes most CTCs, and a minority of escaping cancer cells form metastasis lesions [5]. Since the mid-1980s, it has been evident that the gene expression portrayal within the tumor cells dictates cancer metastasis [6,7]. A limited number of tumor cells acquire metastatic potential through the aberrant expression of several intertwining genes.

Further, besides the set of genes of a positive regulator of dissemination, the genes for metastasis suppression also need to be inactivated for tumor cells to disseminate [8,9].

Heterogeneic clonal evolutions, interacting host factors, and consistent genetic adjustment are required to complete metastasis.

Several decades of research have identified several genes are associated with distant organ-specific metastasis [8,9,10,11]; still, it is not enough to explain the molecular mechanisms of organ-specific metastasis. We compared the gene expression profiles of poorly metastatic mouse breast cancer 4T1 cells with a highly metastatic variant 4T1.2 cells. 4T1.2 cells were identified from the 4T1 breast cancer bone metastasis by several rounds of breast implantation and resection of primary tumors using immune-competent mouse models [12,13]. We have performed transcriptomic analyses to compare gene expression profiles between TNBC metastatic variant 4T1.2 and parental 4T1 cells. Our goal was to identify and validate the set of genes of 4T1.2 tumors are associated with distant metastasis in the mouse model.

We use syngeneic implantation/resection distant metastasis models to validate the gene expression data and compare the gene expression at the primary site vs. distant metastatic sites. Using concurrent clinical and molecular information from a large cohort of breast cancer patients, we developed an individual highly overexpressing gene score associated with distant survival metastasis in multiple cohorts.

We hypothesized that altered expression of genes between primary tumors vs. distant sites might reflect breast cancer’s overall aggressiveness, and those genes could be potential targets for distant organ metastasis.

## 2. Results

### 2.1. Identifying Potential Gene Signatures in TNBC Metastatic Variants of Breast Cancer Cells

**Figure 1:**
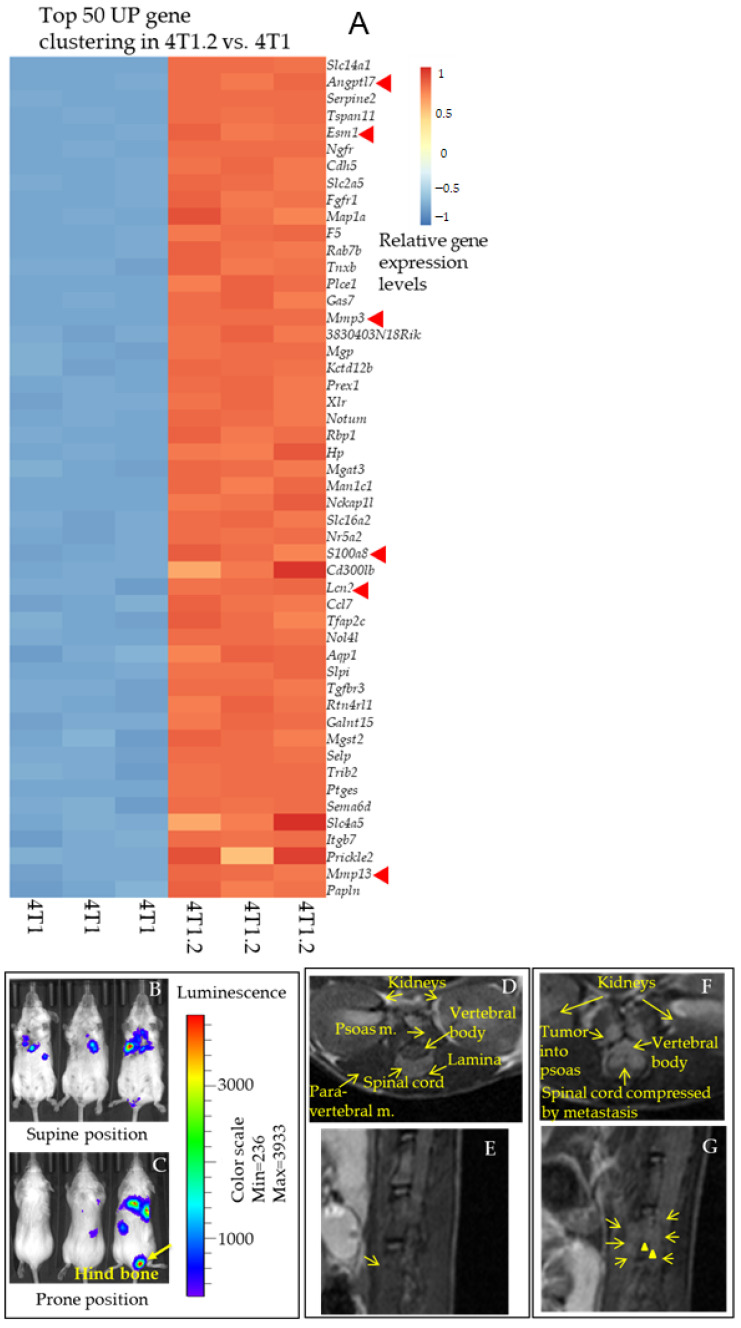
Proangiogenic and pro-inflammatory secretory protein-encoding genes are upregulated in metastatic variant 4T1.2 vs. parental 4T1 cells. (**A**) RNA-Seq heat map for 50 upregulated genes in 4T1.2 vs. 4T1 cells. Genes indicated in bold red arrow revealed a fold change (>6-fold vs. 4T1) is the mediator protein-encoding genes are elevated in the 4T1.2 cells. Of note, there are 3250 genes altered (upregulated and downregulated; padj < 0.05; fold change (FC) > 2) in these cell lines. In total, 1729 genes are upregulated in 4T1.2 cells, whereas 1521 genes are downregulated in 4T1.2 cells vs. 4T1 cells. (B-C) On day 17 of the inoculation of cancer cells in the implantation/resection model, IVIS live images of Balb/c mice bearing metastatic 4T1.2-Luc+ breast cancer cells at primary and metastatic sites, supine (**B**), and prone (**C**) positions are shown. Arrow indicates hind bone 4T1.2-Luc+ metastasis. Representative mice images are shown (*n* = 5). (**D**–**G**), MRI of 4T1.2-Luc+ spine bone metastasis in vivo. On the day of sacrifice (day 17) of 4T1.2-Luc+ metastatic cancer-bearing Balb/c mice, an MRI scan was performed for the mice, and representative images were shown. Top left (**D**) and right (**F**), two representative T2-weighted transverse slice images of Balb/c mice with 4T1.2-Luc+ metastatic cancer. Spine metastasis is not visible in Figure D, whereas in Figure 1F, tumor cells infiltrate into the psoas, tumor extended into the spinal canal, clinically it is called “Metastatic Epidural Spinal Cord Compression (MESCC)”. Bottom left (**E**) and right (**G**), two representative spine images visualized by the T2-weighted MRI, sagittal projection. Arrows indicate spine bone metastasis (**G**). Data are representative of *n* = 5 mice.

To comprehensively investigate genes playing a role in breast cancer metastatic phenotypes, we have performed RNA-seq analysis of 4T1 and derivative cell line 4T1.2 with varied metastatic abilities to lung, bone, and spine. The cells are grown in vitro, and RNA was isolated to perform RNA-seq analysis to understand how these two cell lines are different in terms of their gene expression, explaining the metastatic nature of 4T1.2 cells. We found a cluster of top 50 genes differentially expressed between 4T1 and 4T1.2. (Figure 1A and Figure S1). As shown in Figure 1A, several pro-angiogenic and pro-inflammatory secretory genes are highly upregulated in 4T1.2 cells compared to 4T1 parental cells. Interestingly, genes namely Angiopoietin related protein 7 (ANGPTL7) [Log2fold change (10.042)], Serpine2 [Log2fold change (8.99)], Teraspanin11 [Log2fold change (8.9)], Endothelial cell-specific molecule 1 (ESM1) [Log2fold change (8.88)], Cadherin 5 (CDH5) [Log2fold change (8.59)], Matrix metalloproteinase protein 3 (MMP3) [Log2fold change (7.8)], S100A8 [Log2fold change (6.9], and Lipocalin2 (LCN2) [Log2fold change (6.6)] are the most important genes that are downregulated in 4T1 while upregulated in 4T1.2. However, claudin4 [Log2fold change (−10.6)], Epithelial splicing regulated protein1 [Log2fold change (−10.4)], Fermitin family homolog1 Met transcriptional regulator [Log2fold change (−10.2)] are downregulated in 4T1.2 compared to 4T1 cell lines (Figure S1). This heatmap data analysis gives us an allusion that although 4T1.2 is a metastatic derivative of 4T1, they have differential gene expression levels. We wanted to validate some of our gene expression RNA-seq data of 4T1.2 cells using the metastatic syngeneic mouse tumor model. As expected, 4T1.2-Luc+ cells show metastatic lesions in the lymph node (LN), lung, spine, and hind bones after primary tumors were resected, identified by the IVIS imaging signal on both ventral and dorsal sides (Figure 1B,C). Further, MRI analysis of those mice (Figure 1D–G and Figure S1B–I, representative MRI images, upper and lower panels) showed cancer cell colonization in the spinal cord (Figure 1F,G and Figure S1F,G), lungs (Figure S1B–E) explaining recurrence and metastatic spread of the 4T1.2-Luc+ syngeneic tumor model. 

### 2.2. ANGPTL7 Secreted Angiogenesis-Related Protein-Encoding Gene Alteration in Breast Cancer Distant Metastasis

Profiling of human tissue can distinguish the gene expression between normal and tumor tissue. However, there could be an interference of therapies on the alteration of gene expressions. Gene signatures derived from treatment-naive mouse metastatic models could predict the impact of gene expression on cancer prognosis. Our goals are to (1) identify genes that are responsible for breast cancer organotropic distant metastases, (2) validate our metastatic 4T1.2 cell line RNA-seq data using breast cancer metastatic recurrence mouse models, and (3) link those altered genes with the breast cancer patient survival using publicly available breast cancer metastasis cohorts. Based on our RNA-seq analysis, ANGPTL7 is one of the most upregulated genes in 4T1.2 cells. Angiopoietin-like (ANGPTL) proteins belong to a family composed of eight members (from ANGPTL1 to ANGPTL8) [14]. ANGPTL proteins are secreted proteins showing structural similarity to members of the Angiopoietin (ANG) family, with a coiled-coil domain at the N-terminus and a fibrinogen-like domain at the C-terminus. Several ANGPTL proteins potently modulate angiogenesis [15,16,17,18]. ANGPTL7 protein was initially found in the human corneal stroma, trabecular meshwork, and sclera. Protein levels of ANGPTL7 are over-expressed in glaucomatous aqueous humor [19,20,21]. ANGPTL7 has been emerging as an important pro-angiogenetic factor involved in inflammation and tumor progression, and metastasis [21,22,23].

**Figure 2:**
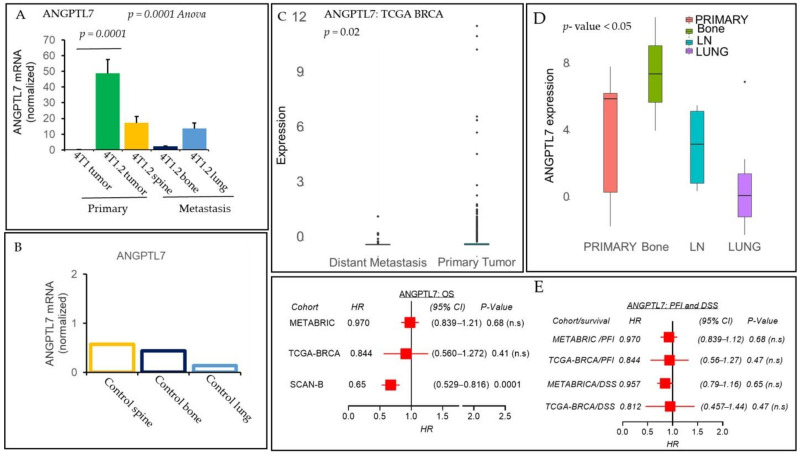
ANGPTL7 is downregulated at the metastatic sites vs. primary tumor and positively impacts breast cancer patients’ survival. (**A**) ANGPTL7 mRNA levels, determined by qPCR analysis, were compared between primary tumors of 4T1 vs. 4T1.2 cells (*n* = 3/group). Metastatic lesions of 4T1.2 cells at distant sites such as the spine, bone, and the lung, were used for ANGPTL7 mRNA levels using qPCR analysis (*n* = 3). (**B**) ANGPTL7 expression levels were also estimated in the control mice (*n* = 3) tissues of the spine, bone, and lung. ANGPTL7 mRNA levels were normalized with GAPDH. All the qPCR experiments were repeated at least twice to obtain consistent results. Data are mean ± SEM, one-way ANOVA *p* = 0.0001, Tukey’s post hoc test, *p* < 0.05, *n* = 3. (**C**) A boxplot shows a high expression score of the ANGPTL7 gene in breast cancer patients of TCGA-BRCA cohort with primary tumors (*n* = 979) vs. patients with metastases (*n* = 64). The mean bar value for distant metastasis is −0.16641875, and the primary tumor is −0.07244. Student’s *t*-test, *p* = 0.05. (**D**) Boxplots show a high ANGPTL7 expression score of primary tumors vs. metastasis to bone, LN (lymph node), or the lung in GSE110590 cohort of 16 patients [25], boxplots analyses, Kruskal–Wallis test (non-parametric method), *p* < 0.05; Tukey’s *t*-test with medians and inter-quartile ranges, *p* < 0.05, as a significant difference. (**E**) Patients with a high ANGPTL7 expression score and association of survival in three breast cancer cohorts. Overall survival (OS) in METABRIC, TCGA-BRCA, and SCAN-B cohorts (left) or progression-free survival (PFI) and disease-specific (DSS) survival (right) in METABRIC and TCGA-BRCA cohorts along with hazard ratios (HR) and their 95% confidence intervals (CI) and *p*-values are shown. Note: Only SCAN-B OS data for a high expression score of ANGPTL7 is significant (HR = 0.65, *p* = 0.0001).

Therefore, to validate our RNA-seq data of ANGPTL7, a qPCR analysis was performed using primary breast tumors of 4T1 and 4T1.2 compared with the expression level at the metastatic sites, e.g., spine, bone, and lung using 4T1.2 syngeneic recurrence/metastasis mouse model. As shown in Figure 2A, ANGPTL7 expression was significantly higher in 4T1.2 primary breast tumors than 4T1 primary breast tumors (*p* = 0.0001), suggesting that cells maintained the increased expression of ANGPTL7 when they formed breast tumors in mice. In order to understand that ANGPTL7 contributed to establishing metastatic lesions in distant organs, we have performed qPCR analyses to examine and compare the expression level of ANGPTL7 in the metastatic lesions isolated from the spine, bone, and lung. Our qPCR data showed that ANGPTL7 expression was reduced in metastatic lesions in the spine, bone, and lung. However, reduced expression of ANGPTL7 at the distant sites’ is significantly higher (Figure 2A) than the normal spine, bone, and lung endogenous tissue-specific expression (Figure 2B), suggesting truncated expression ANGPTL7 are linked to breast cancer metastasis. Although ANGPTL7 is overexpressed in cancer, as shown in Figure 2C, its expression levels were significantly reduced in the distant metastasis group of patients compared to patients with primary tumors in the TCGA-BRCA cohort [24]. In contrast, the ANGPTL7 expression score for the TCGA-BRCA cohort using a paired comparison of absolute log2 fold change (FC) gene expression values with Welch’s *t*-test suggested that ANGPTL7 was significantly higher in adjacent normal tissue compared to the tumor (*p* < 0.00001).

We also examined the ANGPL7 expression levels in primary comparing with bone, lymph node (LN), and lung-metastatic tumors of the breast cancer in the GSE110590 cohort [25]. As shown in Figure 2D, expression levels of ANGPTL7 were significantly higher in bone, LN than the primary tumor, with a tendency of reduced expression in lung metastasis. To comprehend the prognostic value of the ANGPTL7 expression using Kaplan–Meier analysis of OS in SCAN-B cohort having data of 3273 breast cancer patients [26]. We chose to divide the patients into ANGPTL7 high- and low-expression groups using the top one-third of the score as the cutoff. Data showed in Figure 2E (right panel), the hazard ratio (HR) for progression-free survival (PFI) or disease-specific survival (DSS) for METABRIC and TCGA-BRCA with the high expression of ANGPTL7 was not significant.

However, the HR for OS with the ANGPTL7 expression was 0.657 (95% CI = 0.529–0.816; *p* < 0.001) for the SACN-B cohort, suggesting breast cancer patients with a high ANGPTL7 expression score have better survival (Figure 2E, left panel).

### 2.3. MMP3 Matrix Metalloproteinase Secreted Endopeptidase Protein-Encoding Gene Inversely Linked in Breast Cancer Distant Metastasis

Matrix metalloproteinases (MMPs) belong to a large proenzymes family of secreted proteins. These proteins play a significant role in the degradation and remodeling of the extracellular matrix (ECM). MMPs are found overexpressed in a variety of diseases, including cancers. We found MMP3 and MMP13 were upregulated in metastatic 4T1.2 cell lines in our RNA seq data; we pursue determining if MMP3 expression is elevated at the distant metastasis sites in mouse models and could be a useful prognostic marker for patient survival. One of the earlier studies conducted by Lochter et al. discovered the role of MMP3 in cancer metastasis [27]. However, several studies reporting that MMP3 exhibits both tumor-promoting and tumor-inhibiting effects based on its actions on the targeted substrates.

**Figure 3:**
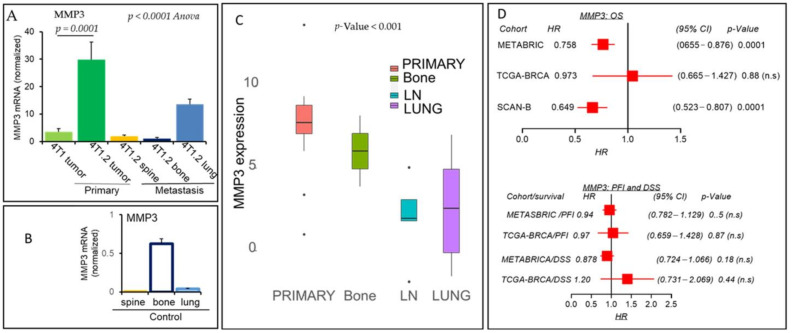
MMP3 is downregulated at the metastatic sites vs. primary tumor and positively impacts breast cancer patients’ survival. MMP3 mRNA levels were determined by qPCR analysis in the primary tumors, metastatic lesions (**A**), and corresponding control tissues (**B**) of mice, as mentioned in Figure 2A. MMP3 mRNA levels were normalized with GAPDH. Data are mean ± SEM, one-way ANOVA *p* = 0.0001, Tukey’s post hoc test, *p* < 0.05, *n* = 3. (**C**) Boxplots show a high MMP3 expression score of primary tumors vs. metastasis to bone, LN, or the lung. Boxplots analyses, Kruskal–Wallis test (non-parametric method), *p* < 0.05; Tukey’s *t*-test with medians and inter-quartile ranges, *p* < 0.05, as a significant difference among groups having different sample sizes. (**D**) Patients with a high MMP3 expression score and association of survival in three breast cancer cohorts. Overall survival (OS) in METABRIC, TCGA-BRCA, and SCAN-B cohorts (upper) or progression-free survival (PFI) and disease-specific (DSS) survival in METABRIC and TCGA-BRCA cohorts along with HR, CI, and *p* values are shown (lower). Note: METABRIC and SCAN-B OS data for a high expression score of MMP3 is significant (HR < 1, *p* = 0.0001).

For example, the interaction between MMP3 and connective tissue growth factor results in the release of angiogenesis-promoting factors [28]. MMP3-mediated cleavage of other growth factors such as heparin-bound epidermal growth factor and transforming growth factor β promotes cancer cell proliferation and epithelial-mesenchymal transition (EMT). In these cases, MMP3 exhibits its tumor-promoting effects. As per our animal experiment data (Figure 3A), the expression of MMP3 was higher in primary tumors of 4T1.2 than primary tumors of 4T1 in our metastatic mouse model (*p* = 0.0001). However, MMP3 gene expression levels were reduced in metastatic lesions of the spine, bone, and lung (Figure 3A). MMP3 expression at the lung’s metastatic lesion is higher (Figure 3A) than the expression of control mouse lung tissues (Figure 3B). In contrast, TCGA-BRCA cohort [24] gene expression analysis revealed that MMP3 expression levels were higher in adjacent normal tissues than patients’ tumors. Further analysis using breast cancer metastasis GSE110590 cohort [25] showed that MMP3 was significantly altered in the bone, LN, and lung metastasis tissues compared to the primary tumor, with a propensity of reduced expression in LN and lung metastasis (Figure 3C). The prognostic value of the MMP3 was also tested by Kaplan–Meier analysis of OS using the SCAN-B cohort of 3273 breast cancer patients [26] and validated using the METABRIC cohort of 1904 breast cancer patients [29] or TCGA-BRCA cohort of 1091 patients [24]. Figure 3D (upper panel) demonstrated that the HR values for OS with the MMP3 expression of high and low-group were less than 1 for both the SCAN-B and METABRIC cohorts (*p* = 0.0001) but not for TCGA-BRCA cohort (*p* = 0.88). These data suggest that breast cancer patients with higher MMP3 expression scores are associated with a better prognosis of breast cancer patients’ survival. However, higher MMP3 expression scores are not associated with or DSS for both the METABRIC and TCGA-BRCA cohorts (Figure 3D, lower panel).

Matrix metallopeptidase-13 (MMP-13) also belongs to the MMP superfamily and is also called collagenase 3. MMP-13 in mice plays a critical role in musculoskeletal development. Mouse MMP-13 is markedly upregulated in the stroma during tumor growth and the progression of breast cancer.

In humans, MMP-13 is highly overexpressed in patients with rheumatoid arthritis [30], osteoarthritis [31], and lymphangiogenesis of multiple myeloma [32]. A similar pattern of elevated MMP13 expression levels was observed in 4T1.2 primary tumors and spine metastasis (Figure S2A–C). However, MMP13 expression scores are not linked with the prognosis of breast cancer patients’ survival using TCGA-BRA, SCAN-B, or METABRIC cohorts. So, we decided not to include the patient’s survival data for MMP13 gene expression.

### 2.4. Positive Association of Lipocalin-2 Secreted Glycoprotein-Encoding Gene and Breast Cancer Distant Metastasis

Lipocalin 2 (LCN2) is a secreted glycoprotein belonging to a lipocalin protein family. LCN2 expression levels are elevated in various human diseases [33,34] and several cancers [35,36], including breast cancer, by inducing the EMT in breast cancer cells [37,38]. We hypothesized that LCN2 is elevated in breast cancer metastasis and is linked with breast cancer aggressiveness, which could be a prognostic factor for the patient’s survival.

**Figure 4:**
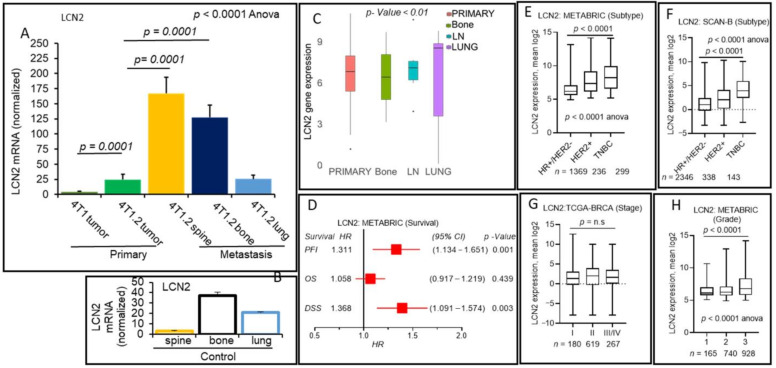
LCN2 is upregulated at the metastatic sites (spine and bone) vs. primary tumor and an independent prognostic factor for breast cancer patients. (**A**,**B**) qPCR analyses were performed to validate RNA-seq data between 4T1 vs. 4T1.2 cell lines using primary tumors. LCN2 mRNA normalized levels were determined in 4T1.2 primary tumors vs. metastatic lesions in the spine, bone, and lung (**A**). LCN2 mRNA normalized levels were also determined in the control tissues isolated from the spine, bone, and lung (**B**). mRNA levels were normalized with GAPDH, as described before. Data are mean ± SEM, one-way ANOVA *p* < 0.0001, Tukey’s post hoc test, *p* < 0.05, *n* = 3. (**C**) Boxplots show a high LCN2 expression score of primary tumors vs. metastasis to bone, LN, or the lung. Boxplots analyses, the Kruskal–Wallis test (non-parametric method), *p* < 0.05, suggested an altered expression of LCN2. (**D**) Patients with a high LCN2 expression score and association of survival of breast cancer patients for the METABRIC cohort. LCN2 high vs. low expression scores survival rates (PFI, OS, or DSS) for the patients of METABRIC cohort and HR, CI, and *p* values are shown (**D**). Boxplots of the LCN2 high expression score by immunohistochemistry (IHC) determined subtype in the METABRIC (**E**) and SCAN-B cohorts (**F**). Similar data were obtained from the TCGA-BRCA cohort. All boxplots are of Tukey type, and boxes depict medians and inter-quartile ranges. One-way ANOVA was used to calculate the *p*-value. Boxplots of high expression of LCN2 score of tumors of different American Joint Committee on Cancer (AJCC) stages (**G**) and Nottingham pathological grades (**H**) are shown for the METABRIC cohort. Similar data obtained from the TCGA-BRCA cohort. One-way ANOVA and Tukey’s tests were used to calculate *p* values.

In compliance with our RNA-seq data of cell lines, the LCN2 transcript is elevated significantly (*p* = 0.0001) in the 4T1.2 primary tumor compared to the 4T1 primary tumor in the metastatic mouse model (Figure 4A). It is noteworthy that Bone and Lung have a significant level of endogenous expression of LCN2 (Figure 4B). Furthermore, our data indicated that LCN2 is markedly elevated (6-fold vs. primary tumor) in the distant metastatic sites of the spine (*p* = 0.0001) and bone (0.0001) compared to the primary tumors (Figure 4A) in addition to the endogenous expression level of control spine and bone tissues of mice (Figure 4B). We found a reduced expression level of LCN2 in the primary tumor of breast cancer patients vs. adjacent normal tissues by analyzing gene expression data of the TACGA-BRCA cohort. Further, we found that LCN2 expression levels are altered in distant sites compared to primary tumors of breast cancer patients’ as revealed from metastatic cohort (GSE110590) data analysis (Figure 4C). We performed survival analysis on the METABRIC data set and used Hazard regression analysis models to evaluate the association of high LCN2 gene expression scores with OS, DSS, and PFI (Figure 4D). In supportive of our hypothesis, higher LCN2 expression was associated with worse DSS (HR 1.36, 95% CI 1.09–1.57, *p* = 0.003), PFI (HR = 1.31, (95% CI = 1.134–1.65, *p* = 0.001), but not for OS (*p* = 0.43) among breast cancer patients of the METABRIC cohort (Figure 4D). Comprehensive analysis of higher LCN2 expression and survival prognosis did not show any prognostic correlation for SCAN-B and TCGA cohort data sets with breast cancer major subtypes (Table S2). Of our interest, we explore further to understand if a higher expression of LCN2 is linked to aggressiveness and advance stage of breast cancer using the METABRIC cohort. Interestingly, we found that the LCN2 expression score was higher in TNBC and HER2+ subtypes, which are known to be more clinically aggressive than hormone receptor (HR+) and HER2− subtype (Figure 4E, *p* = 0.0001, and Table S1A). Similar data were also obtained in the SCAN-B cohort (Figure 4F, *p* = 0.0001, and Table S1A), despite no correlation with survival in this cohort, as mentioned earlier, suggesting LCN2 expression is elevated in more aggressive types of breast cancers. We also hypothesized that the LCN2 gene expression score is associated with aggressive clinical parameters such as pathological grade and cancer stage. Further digging detail into the clinic-pathological variable in the METABRIC cohort, our data explained that the LCN2 gene expression was higher for advanced cancer staging in the METABRIC cohort (Figure 4G and Table S1B), and the trend was observed in the TCGA cohort (Table S1B). LCN2 gene expression score was significantly associated with Nottingham pathological grade (grade 3) in the METABRIC cohort (Figure 4H and Table S1C), and a similar trend was observed in the SCAN-B cohort (*p* = 0.06, grade 3 vs. grade 1 or 2; Table S1C).

Thus, all these results suggested that LCN2 expression is elevated at the distant metastatic sites and may add prognostic value, particularly to Nottingham pathological grade and advanced cancer staging.

### 2.5. S100A8 Secreted Pro-Inflammatory Mediator Encoding Gene Expression Correlates with Breast Cancer Progression and Metastasis

**Figure 5:**
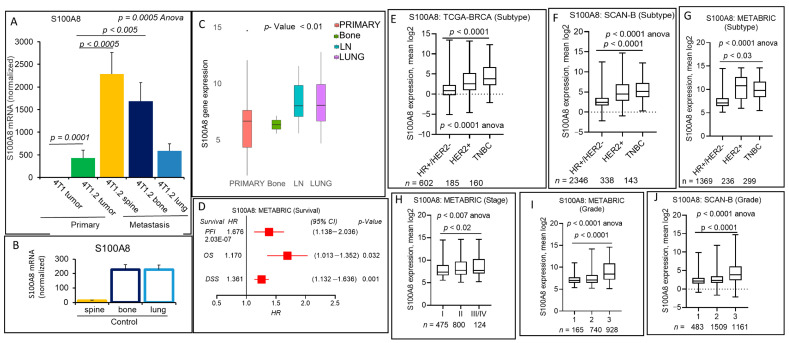
S100A8 is upregulated at the metastatic sites (spine and bone) vs. primary tumor and an independent prognostic factor for breast cancer patients. (**A**,**B**) qPCR analyses for S100A8 gene. (**A**) Elevated levels of S100A8 mRNA in the primary tumors of 4T1.2 vs. 4T1 tumors were confirmed using qPCR analysis. S100A8 mRNA levels were also measures at the distant metastatic lesions of the spine, bone, and lung (**A**). S100A8 mRNA levels were also measured in the corresponding normal tissues of the control mice (**B**). mRNA levels were normalized with GAPDH. Data are mean ± SEM, one-way ANOVA *p* < 0.0006, Tukey’s post hoc test, *p* < 0.05, *n* = 3. (**C**) Altered expression of the S100A8 mRNA was also observed in patients with primary tumors vs. metastatic tumors, as shown by boxplots. (**D**) Patients with a high S100A8 expression score and association of survival (PFI, OS, and DSS) in the breast cancer cohort. Patient survival (PFI, OS, and DSS) data based on the high expression of S100A8 in the METABRIC cohort of breast cancer patients and HR, CI, and *p* values are shown (**D**). Boxplots of the S100A8 high expression score by immunohistochemistry (IHC) determined subtype in the TCGA-BRCA (**E**), SCAN-B (**F**), and METABRIC cohorts (**G**). All boxplots are of Tukey type, and boxes depict medians and inter-quartile ranges. One-way ANOVA was used to calculate the *p*-value. Boxplots of high expression of S100A8 score of tumors of different AJCC stages (**H**) for METABRIC cohort and Nottingham pathological grades are shown for the METABRIC (**I**) and SCAN-B cohorts (**J**). One-way ANOVA and Tukey’s tests were used to calculate *p* values.

Among the top 50 genes, those were upregulated in 4T1.2 cell line RNA-seq analysis, S100A8 is another secreted molecule. S100 groups of proteins are cytosolic calcium-binding families of protein that play important roles in inflammation and cancer [39,40,41]. S100A8 expression levels are found elevated in several cancers [42,43,44], including breast cancer [45], which could be a biomarker in solid tumors. As S100A8 functions to maintain immune homeostasis [40,46], we first look at the endogenous expression of S100A8 in the control bone, spine, and lung of the mice. Unsurprisingly, qPCR data demonstrated that control bone and lung have a higher expression (200-fold normalized value with GAPDH) than the spine (Figure 5B).

Interestingly, in conformity with our cell line RNA-seq data, 4T1.2 primary breast tumors showed increased expression of S100A8 compared to 4T1 primary breast tumors (Figure 5A). Surprisingly, S100A8 expression levels are significantly elevated in the spine and metastatic bone lesions (*p* values are 0.0005 and 0.005, respectively). Lung metastatic lesion showed no further increased expression of S100A8 compared to 4T1.2 primary breast tumors (Figure 5A). After analyzing TCGA-BRCA and breast cancer metastatic cohort (GSE110590) gene expression data, we found that S100A8 transcripts are altered in metastatic sites with a higher expression tendency in metastasis (Figure 5C). Given the strong association between high expression of S100A8 in the primary and metastatic lesion in animal models, we hypothesized that breast cancer with a high S100A8 expression score is also associated with a worse prognosis of survival and associated with clinical parameters with aggressive breast cancers. Our extensive analysis divulged that a high expression score of S100A8 was associated with worse survival (PFI, OS, and DSS; *p* < 0.05) of breast cancer patients in METABRIC cohorts (Figure 5D and the OS data were validated in the SCAN-B cohort of 3273 patients (HR = 1.799, CI 95%; 1.451–2.231, log-rank *p* = 0.0000001) (Figure not included)). However, no significant association was found between S100A8 gene expression and patient survival in major breast cancer subtypes (Table S2).

Further exploration using TCAGA-BRCA, SCAN-B, and METABRIC cohorts data analysis, revealed that a high S100A8 expression score was associated with the aggressive subtype TNBC or HER2+ breast cancers compared to the HR+ or HER2− breast cancer subtypes (Figure 5E–G and Table S1A). As shown in Figure 5H–J and Figure S1B,C, using multiple cohorts data analyses, we have also found that a high S100A8 expression score was associated with higher stages (II/IV) and higher pathological grade (grade 3) of breast cancer patients.

Together, these results suggest that S100A8 is one of the important mediators associated with aggressive breast cancer metastasis and a prognostic factor for worse survival of breast cancer patients.

### 2.6. ESM-1 Secreted Endothelial Proteoglycan Encoding Gene Expression Is Associated with Poor Prognosis of Aggressive Subtypes of Breast Cancers

**Figure 6:**
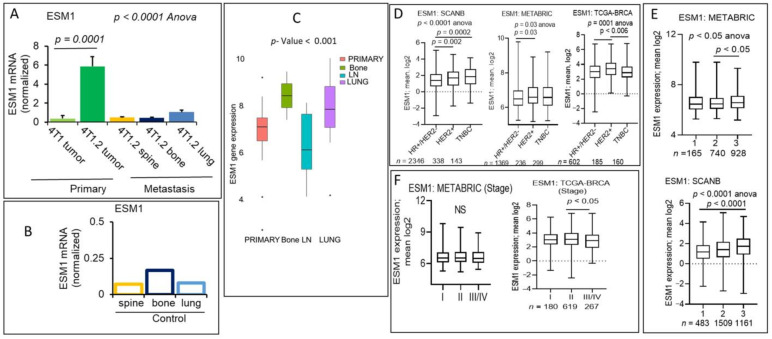
Expression of ESM-1 is downregulated in the 4T1.2 metastatic lesions vs. primary tumors and an independent prognostic factor for aggressive breast cancer subtypes. (**A**,**B**) ESM-1 gene expression levels were analyzed by qPCR in the primary tumors, metastatic lesions (**A**), and control mice tissues (**B**), as labeled. ESM-1 mRNA levels were normalized with the GAPGH. Data are mean ± SEM, one-way ANOVA *p* < 0.0001, *n* = 3. (**C**) Boxplot data showed that ESM-1 mRNA levels were altered in primary tumors vs. metastatic tumors of breast cancer patients, Kruskal–Wallis test (non-parametric method), *p* < 0.05. (**D**) Boxplots of the ESM-1 high expression score by immunohistochemistry (IHC) determined subtype in the SCAN-B (left), METABRIC (middle), and TCGA-BRCA cohorts (right). Boxplots of high expression of ESM-1 score of tumors of different AJCC stages (**F**) for METABRIC (left panel) and TCGA-BRCA cohorts (right panel) and (**E**) Nottingham pathological grades in METABRIC (upper panel) and SCAN-B cohorts (lower panel). One-way ANOVA and Tukey’s tests were used to calculate *p* values.

Endothelial cell-specific molecule-1 (ESM-1) is a 50-kDa proteoglycan secreted by diverse endothelial cells (ECs) [47]. The angiogenic growth factor VEGF or the pro-inflammatory cytokine TNFα upregulates the expression of ESM-1 [48,49,50]. A variety of studies have reported that a high level of ESM-1 secretion is found in several cancers [51,52,53,54], including TNBC [55], and ESM-1 levels have been implicated to play a role in tumor metastasis, migration, and vascular invasion in human cancers by regulating the expression of MMPs [53,56,57]. However, the expression levels of ESM-1 at the metastatic sites, its role in aggressive breast cancers, and whether it could be a prognostic factor of aggressive types of breast cancer were not investigated before. Consistent with our RNA-seq data, metastatic 4T1.2 cell primary tumors have almost six-fold elevated ESM-1 gene expression compared to the 4T1 primary tumors (Figure 6A).

Further, ESM-1 expression levels were reduced at the metastatic sites such as the spine, bone, and lung; however, its levels at the distant metastatic sites were higher (Figure 6A) than those found at corresponding control tissues of mice (Figure 6B). The ESM-1 expression levels were found elevated in the primary tumors compared to the adjacent control tissues of breast cancer patients of TCAGA-BRCA cohort (*p* < 0.000001, *n* = 1091) and showed diverse distribution at the metastatic sites compared to primary tumors of breast cancer patients of the metastatic cohort (Figure 6C). To establish the prognostic value of ESM-1 expression, we analyzed survival data in several breast cancer cohorts and represented our analysis in table format (Table 1). Deep analysis of survival in different cohorts implies, indeed, ESM-1 expression was linked with poor survival (OS, and DSS, and PFI) of breast cancer patients (Table 1). We hypothesized that ESM-1 is a prognostic factor associated with the aggressive breast cancer progression, higher stage, and pathological grade of breast cancer. As shown in Figure 6E,F and Table S1B,C, a higher expression score of ESM-1 was associated with more metastatic (stage III/IV) (Figure 6F and Table S1B) and higher pathological grade (grade 2/3) (Figure 6E and Table S1C) of breast cancers. We have also determined the clinical parameters associated with the higher expression score of the ESM-1 gene. Data analysis using multiple cohorts has suggested that a higher expression score of ESM-1 was associated with the more aggressive subtype TNBC or HER2+ compare to the HR+ or HER2− subtypes of breast cancers (Figure 6D and Table S1A). We further extend our analysis by comparing the higher expression of ESM-1 associated with TNBC patients and survival in three breast cancer cohorts (Table 2 and Table S2). Data explained that the TNBC subtype with high expression of ESM-1 had worse survival (DSS, HR 1.67, *p* = 0.02) and also worse survival for HR+/HER2− patients (OS, DSS, or RFS) compared to HER2+ subtype in the METABRIC cohort (Table 2 and Table S2). ESM-1 high expression was also linked with worse survival (DSS or PFI) of HER2+ vs. other subtype patients in the TCGA-BRCA cohort, associated with the poor OS survival of HER2+ patients of SCAN-B cohort (Table 2 and Table S2).

Together, data suggested that the ESM-1 gene is overexpressed in metastatic aggressive subtypes of breast cancers and could be an independent prognostic factor. 

## 3. Discussion

Breast cancer metastasis is a complex process that requires alteration of gene expression allowing tumor cells to escape from the primary tumor site [58]. Among several molecular mechanisms that enhance the metastasis process, the secretion of mediator molecules by tumor cells may facilitate evasion from the immune system’s detection. Primary tumor-derived secretory factors impact the cancer cells in the tumor microenvironment [59].

Metastatic cells are challenged to acquire a specific characteristic for the aggressive phenotype [60]. Indeed, these secretory molecules may have roles to impact primary tumor cells and influence tumor microenvironment to create favorable conditions for metastases to occur. Therefore, there is a selection of specific phenotypes in cancer cells due to interaction with the tumor microenvironment that evolves with the primary tumor during tumor progression.

So far, to our knowledge, there is no such study where there is a comparison of gene expression between primary tumors and how each particular gene expression changes at the metastatic sites such as the spine, bone, or the lung. To identify genes for metastatic breast cancer, we compared the gene expression of a poorly metastatic 4T1 cell line and its variant, 4T1.2, which was selected in vivo after multiple implantation/resection, a higher tendency for spontaneous metastatic spread using syngeneic mouse models [12,13]. Our study aimed to validate the overexpressed mediator encoding genes of 4T1.2 in the implantation/resection metastasis mouse model and develop a prognostic biomarker for the occurrence of distant metastasis in breast cancer based on gene expression profiles of bulk tumors.

Interestingly, primary tumor expressing ANGPTL7, MMP3, LCN2, S100A8, and ESM-1 levels that were upregulated in 4T1.2 cells was strongly associated with breast cancer patients’ survival outcomes. ANGPTL7 is a member of the ANGPTL family, emerging as an important regulator of metastasis development [22]. Although ANGPTL7 is overexpressed in breast cancer [22], the role of ANGPTL7 in breast cancer progression and metastasis is still unclear. Altogether, in the literature, the angiogenetic role of ANGPTL7 is inconsistent [61]; for instance, some studies reported pro-angiogenetic effect [22] while others suggested antiangiogenic activity [62,63]. The function of ANGPTL7 in the tumor microenvironment (TME) may be complex associated with tumor progression and metastasis. Consistent with several reports, our experimental data suggest that cancer cells inhibit angiopoietin-like protein gene expression to progress metastasis in breast cancer.

Furthermore, the cohort data, suggesting that a high expression score of ANGPTL7 was associated with a better OS. Coequally, MMP3 levels were downregulated in distant metastatic sites such as the spine, bone, and lung in the implantation/resection mouse model. Several studies have demonstrated the involvement of MMPs to promote angiogenesis and metastasis [64,65,66]. By using web-based KM Plotter [67], it has been shown that patients expressing higher levels of MMP3 had a significantly poorer outcome for distant metastasis-free survival (DMFS) [68]. Our further interrogation using SCAN-B and METABRIC cohorts of breast cancer patients suggested that a high MMP3 expression score was associated with the better OS of patients (HR < 1, *p* = 0.0001). One of the possible reasons could be that angiogenesis secretory factors ANGPTL7 and MMP3 are able to promote vascularization by inducing angiogenesis and thereby overexpressed in several cancers. Henceforth, more vascularization might help better drug delivery, which might be the reason for better survival. Our 3rd highly upregulated secretory mediator LCN2, is elevated in varieties of cancers, and is associated with breast cancer progression [36,37,38].

Heterogeneous expression of LCN2 was reported in patients with primary breast cancer. Notably, a significant correlation between LCN2 expression with other markers including ER− negative or progesterone receptor (PR)-negative status has been reported in breast cancer [69,70,71]. Previous reports using web-based KM plotter database data analysis suggested that LCN2 expression predicts poor clinical outcome in TNBC [71]. LCN2 has also been revealed to significantly enhance VEGF-induced angiogenesis in a mouse model [72]. To establish clinical relevance, we investigated further using larger cohorts SCAN-B and METABRIC of breast cancer patients data analysis suggested that LCN2 expression score is high in aggressive breast cancer HER2+ or TNBC, but not linked with any survival outcome of these subtypes. Kurozumi, S., et al. [73] reported that subcellular localization of LCN2 expression is important to controlling breast cancer progression. Loss or reduced expression of nuclear LCN2 is related to the aggressive nature and poor outcome in breast cancer. Notably, other studies have proposed that the high cytoplasmic expression of LCN2 is associated with the decreased disease-free survival in patients with invasive breast cancer [70]. The tumor microenvironment controls the LCN2 function through autocrine system of cancer cells via endoplasmic reticulum stress-dependent and independent mechanisms. LCN2 can be released extracellularly and also internalized through its receptor to control cellular function promoting cell survival [74]. These complex mechanisms may be responsible for the clinicopathologically discrepancy observed with the expression of LCN2 and Survival. S100A8 is a secreted inflammatory mediator, is increasingly recognized as a biomarker in many solid tumors, including breast cancers [45,75]. One previous study uncovered that Plasma samples of preclinical mice model compared with control showed significantly upregulated protein expression of S100A8, S100A9 along with LCN2 [76]. In another study, LCN2 gene expression was upregulated in S100A8 and S100A9-stimulated colon cancer cells compared to non-stimulated cells [77].

These findings, along with our data, suggest that LCN2 expression might be involved in metastases regulation through reciprocal interaction with S100A8 proteins. Several studies strongly suggest that S100A8 is expressed by cancer cells as well as by infiltrating immune and myeloid cells [78]. Since S1008A could be upregulated by different conditions such as oxidative stress, cytokines, and growth factors in many types of cells, in agreement, we found that control bone and lung have a high expression compared to the spine.

ESM-1 endothelial secreted mediator is elevated in a variety of cancers, including breast cancers [55], and it has been implicated in playing a role in tumor metastasis in other cancers. However, its role in breast cancer metastasis and if it could be a prognostic factor for more aggressive subtypes of breast cancer was not known. ESM1 expression that was elevated in the 4T1.2 cells and primary tumors was further downregulated in distant sites such as the spine, bone, and the lung in our implantation/resection metastasis mouse model. Using multiple larger cohorts of breast cancer patients (METABRIC, SCAN-B, and TCGA-BRCA), we have demonstrated that the high expression score of ESM-1 instead exhibits as an independent prognostic factor for worse survival of breast cancer patients. Based on our hypothesis that ESM-1 is an important prognostic factor for aggressive subtypes of breast cancers, we found that a high expression score of ESM-1 was associated with a higher grade and higher stages of breast cancer patients. High expression of ESM-1 was also associated with TNBC, HER2+, in some cases, HR+/HER2− breast cancer patients’ survival outcomes.

These results indicate that mediators encoding genes with prognostic and predictive scores may guide further to understand the clinical importance and design new drugs that target secretory mediators in breast cancer distant metastasis, explicitly in aggressive subtypes TNBC or HER2+ breast cancer.

In conclusion, our results suggest that dissimilarly expression of few mediator encoding genes ANPTl7, MMP3, S100A8, LCN2 ESM1 in primary versus distant metastasis organs and their prognostic and predictive scores in breast cancer cohort may be a helpful to predict future metastasis. Our experimental animal model explained that targeting single gene in primary tumors may not always be beneficial to inhibit metastasis as some of the downregulated genes in the primary tumor are differentially expressed to establish the tumor in distant organ.

## 4. Materials and Methods

### 4.1. Chemicals and Reagents

Cell culture medium, α-MEM (Minimum Essential Medium α), trypsin, penicillin-streptomycin, Phosphate Buffered Saline (PBS), sodium pyruvate and D-luciferin were purchased from Thermo-Fisher Scientific (Waltham, MA, USA). Fetal bovine serum (FBS) was purchased from Peak Serum (Peak Serum, Wellington, CO, USA).

### 4.2. Cell Culture

The 4T1-Luc+ mouse breast cancer cell line and the 4T1.2-Luc+ metastatic variant of 4T1 parental cell line [12] were kindly provided by Prof. Cheryl L. Jorcyk of Boise State University, Department of Biological Sciences, Biomolecular Sciences Program, 1910 University Drive, Boise, ID, 83725, USA. Both the cell lines were cultured and maintained in α-MEM supplemented with 10% fetal bovine serum, 1 mM Penicillin/Streptomycin, and 1 mM sodium pyruvate at 37 °C in 5% CO_2_ and 95% humidity, as mentioned before [79,80,81].

### 4.3. RNA Preparation and RNA-Seq

4T1-Luc+ and 4T1-Luc+ cell lines were used in triplicates for RNA-seq analysis. The cell lines were cultured in a full-serum medium, as mentioned above, and harvested at sub-confluence (60% confluence) for RNA isolation by using a total RNA purification kit with a DNAse treatment step (Qiagen, Valencia, CA, USA). The total RNA quality was evaluated using the Agilent 2100 bioanalyzer (Agilent, Palo Alto, CA, USA) with the RNA 6000 Nano LabChip kit. RNA-seq libraries were prepared using TruSeq Stranded Total RNA Library Prep Gold kit (Illumina, San Diego, CA, USA). The quality of the libraries was validated by assaying using TapeStation D1000 ScreenTape (Agilent) and Library Quantification Kit (Kapa Biosystems, Wilmington, MA, USA). All the top quality libraries were sequenced together on an Illumina HiSeq 2500 instrument using HiSeq Rapid Cluster Kit v2—Paired-End and Rapid SBS Kit v2 reagents to obtain paired reads of 100 bases. Casava software (version 1.8.2, Illumina Inc, San Diego, CA, USA) was used to demultiplex the sequencing data. An average of 100 million sequence read-pairs was obtained for each sample. Raw read data were filtered and mapped in a splicing-aware manner with the data processing using TopHat2 software [82]. Correct read alignment across splice junctions of the raw data was performed by Gencode v25 gtf [83]. HTSeq framework [84] was used for gene-level mapped read count values of the RNA-seq data. Finally, the read count data were normalized, and log2 fold changes were estimated for further analyses with DESeq2 [85].

### 4.4. Gene Expression Analyses

The DESeq2 Bioconductor package for R was used for differential gene expression analyses. Gene levels were considered as differentially expressed based on the expression levels changes of absolute log2 fold-change (FC) > 1.2 and false discovery rate (FDR) < 0.05 after adjustment for multi-testing by Benjamini–Hochberg method. The Benjamini–Hochberg method was used to calculate the FDR (<0.05) as a cutoff to identify the candidate genes for multiple testing adjustments,

### 4.5. Quantitative-Real-Time PCR (qRT-PCR)

According to the manufacturer’s instructions, as described before [79,80,81,86], cDNA was synthesized from DNase pre-treated 1 μg RNA using the SuperScript cDNA Synthesis kit (Life Technologies, Carlsbad, CA, USA). The PCR reaction was performed by the thermal cycler (Bio-Rad, Hercules, CA, USA) using SYBR Green qPCR SuperMixes with PCR primers for the mouse (ANGPTL7: F- TGACTGTTCTTCCCTGTACCA, R- CAAGGCCACTCTTACGTCTCT; MMP3: F- ACATGGAGACTTTGTCCCTTTTG, R- TTGGCTGAGTGGTAGAGTCCC; LCN2: F- TGGCCCTGAGTGTCATGTG, R- CTCTTGTAGCTCATAGATGGTGC; S100A8: F- AAATCACCATGCCCTCTACAAG, R- CCCACTTTTATCACCATCGCAA; ESM-1: F- CTGGAGCGCCAAATATGCG, R- TGAGACTGTACGGTAGCAGGT; GAPDH: F- TGGATTTGGACGCATTGGTC, R- TTTGCACTGGTACGTGTTGAT). All the primer sequences were used from the publicly available mouse primer bank (https://pga.mgh.harvard.edu/primerbank/index.html). Each qPCR sample was run at least in triplicate. The relative level of target genes from each sample was calculated by the 2^−ΔΔCT^ method [87,88] and normalizing to the house-keeping gene GAPDH.

### 4.6. Ethical Statement

All animal methods were approved by the Institutional Animal Care and Use Committee of Roswell Park Comprehensive Cancer Center and were performed in accordance with the relevant guidelines and regulations for the American Association of Laboratory Animal Care. Animals were bred and maintained in a pathogen-free environment, and the RPCCC IACUC approved all procedures with experiments performed under IACUC protocol #1338M.

### 4.7. Animals and Tumor Cell Implantations

Female Balb/c mice, 12 weeks of age, and approximately 20 g/mice were obtained from The Jackson Laboratories (Bar Harbor, ME, USA). 4T1-Luc+ and 4T1.2-Luc+ cells suspended in the culture medium at a concentration of 1 × 10^7^ cells/mL, and 10 μL of this solution were then implanted as described below [89,90]. All cancer cell implantations were performed under isoflurane anesthesia using sterile technique. A 5 mm incision was made medial to the nipple, and a cotton swab was used to expose the mammary gland. The cells were implanted directly into the mammary gland of mice (*n* = 5) under direct vision, using ×10 microscopic magnifications, and the wound was closed with a nylon suture. Xenogen In Vivo Imaging Systems (IVIS^®^) 200 (Version 4.3.1, Alameda, CA, USA) and Living Image^®^ software (Caliper Life Sciences, Hopkinton, MA, USA) was used to quantify the photon/sec emitted by 4T1-Luc+/4T1.2-Luc+ cells after intraperitoneal injection of 200 μL (150 mg/kg) of D-luciferin (Fisher Scientific, Inc., Waltham, MA, USA) for the determination of in vivo tumor burden. After day 3 of the inoculation of cancer cells, mice were randomized based on equal initial low levels of photon counts.

On day 7, IVIS live imaging was performed for the tumor-bearing animals, and no distal metastatic spread of cancer cells was observed. Seven days after implantation, the entire primary tumors were resected from the animals, and the incision was closed. IVIS live imaging confirmed no residual Luc+ cells at the primary sites or any metastatic spread. 4T1.2-Luc+-tumor resected mice were kept for another 10 days for tumor recurrence at distant sites. Distant metastasis of 4T1.2-Luc+ tumors were regularly monitored by IVIS live imaging. On day 17 of inoculation, IVIS live imaging was performed, and mice images were shown, suggesting 4T1.2-Luc+ cells recurred to the distant sites, including the lung and the bone. On day 17, the magnetic resonance imaging (MRI) data confirmed the 4T1.2-Luc+ cancer cell colonization in the spinal cord.

Following instructions, MRI was performed using a laboratory animal MRI scanner (Bruker Medical Inc., Billerica MA, USA) with a magnetic field of 4.7T (Translational Imaging Shared Resource, Roswell Park Cancer Institute). 4T1.2-Luc+ metastatic cancer-bearing mice (*n* = 5) were scanned in multiple stages using transverse and sagittal projections. T2-weighted spin-echo images were acquired for the mice. In T2-weighted imaging, the field of view was 3.2 × 3.2 cm, and the thickness of the slice was 1 mm. The total duration of the MRI scan for each mouse was 20 min. A representative image of the MRI results was shown, suggesting cancer cell recurrence at spine bone. Mice were sacrificed, and metastatic lesions of the spine, bone, and lung were collected based on ex vivo IVIS confirmation. Primary tumors and the metastatic lesions of the distant organs were used for molecular analyses.

### 4.8. Clinical and Gene Expression Data Analyses of Breast Cancer Patient Cohorts

Publicly available clinical parameters and tumor gene expression data for 1091 patients of the TCGA breast cancer (BRCA) Project [24] and 1094 patients for the METABRIC cohort [29] were obtained for analyses from cBioPortal [91]. Tumor gene expression and clinical data of 3273 breast cancer patients were also obtained for the SCAN-B cohort [26]. In terms of survival data, disease-specific survival (DSS), overall survival (OS), and progression-free survival (PFI) were available in TCGA and METABRIC cohorts, and only OS data were available for the SCAN-B cohort. Normalized microarray-based gene expression data (log2-transformed data) for primary tumors and metastatic sites, including the bone lymph node (LN) and the lung, were obtained for analyses from the GEO repository (GSE110590 cohort of 16 patients [25]). Univariate Cox regression analysis was performed using the cohorts to determine which gene’s expression may be an independent prognostic marker for patient survival. Among the top 50 differentially upregulated genes in 4T1.2 cells compared to parental 4T1 cells, ANGPTL7, MMP3, LCN2, S100A8, and ESM-1 were used for analyses for the cohorts. The high/low cutoff for any gene was defined as the top-third vs. the bottom two-thirds within any cohort.

### 4.9. Statistical Analyses

For qPCR data, an unpaired two-tailed Student’s *t*-test was used to compare two groups (using GraphPad Prism version 8.0, San Diego, CA, USA). The levels of mRNAs are expressed as the means ± SEM. We had used a one-way ANOVA test for datasets containing multiple group comparisons and Tukey’s post hoc test for the family-wise error rate comparison. Data plotting were performed using GraphPad Prism 8 or Microsoft Excel (version 16 for Windows, Redmond, WA, USA). Kaplan–Meier method with log-rank test was used for survival analysis. For all analyses, *p* ≤ 0.05 was considered statistically significant.

## 5. Conclusions

We have identified several secreted mediators encoding gene expressions altered in metastatic lesions compared to primary tumors using the syngeneic metastatic mouse model. Enduring clinical relevance, we found that increased expression of these secreted mediators encoding genes have a poor prognosis and can be useful to predict future metastatic potential in distant organs.

## Figures and Tables

**Table 1:** ESM1 expression and survival data in breast cancer whole cohorts.

ESM1					
Cohorts (No. of Patients)	Survival	*p*-Value	HR	Lower 95% CI of HR	Upper 95% CI of HR
TCGA-BRCA (1091)	OS	0.2651561	1.241098	0.847944	1.81654
	DSS	0.0260737	1.802839	1.081905	3.00417
	PFI	0.0404124	1.507879	1.018059	2.23336
SCAN-B (3273)	OS	0.0027642	1.393542	1.122641	1.72981
METABRIC (1904)	OS	0.0002073	1.313813	1.135676	1.51989
	DSS	0.0000432	1.488734	1.229534	1.80257
	PFI	0.0001991	1.421232	1.180513	1.71103

HR—hazard ratio; CI—confidence intervals.

**Table 2:** ESM1 expression and survival data in breast cancer subtypes.

ESM1 Gene						
Breast Cancer Cohorts	Subtypes	Survival	*p*-Value	HR	Lower 95% CI of HR	Upper 95% CI of HR
METABRIC	TNBC	OS	0.0678599	1.405336	0.9650234	2.046551
		DSS	0.0271319	1.671391	1.05759	2.64142
		RFS	0.06816303	1.531018	0.9659142	2.426733
	HER2+	OS	0.2023138	1.304144	0.8662699	1.963351
		DSS	0.4882853	1.170697	0.7486584	1.830649
		RFS	0.4749246	1.177973	0.7512301	1.847131
	HR+/HER2-	OS	0.0084293	1.255485	1.058645	1.488925
		DSS	0.0060594	1.392947	1.098825	1.765796
		RFS	0.0075160	1.357008	1.084659	1.697742
TCGA-BRCA	TNBC	OS	0.5326945	1.329982	0.5391484	3.280826
		DSS	0.3297474	1.753202	0.5614073	5.47502
		PFI	0.690275	1.205813	0.476718	3.049989
	HER2+	OS	0.2602478	1.738325	0.6522829	4.632614
		DSS	0.0343557	6.291787	1.570462	25.20697
		PFI	0.0204855	4.075747	1.366649	12.15506
	HR+/HER2-	OS	0.4214821	1.284964	0.6960809	2.37204
		DSS	0.2860177	1.617747	0.6729301	3.889119
		PFI	0.304155	1.384029	0.7437917	2.575366
SCAN-B	TNBC	OS	0.4688024	1.321067	0.6211149	2.809815
	HER2+	OS	0.0171431	2.245766	1.188923	4.242043
	HR+/HER2-	OS	0.2057267	1.190957	0.9086451	1.560983

## Data Availability

The data presented in this study are available on request from the corresponding author.

## Supplementary Materials

The following are available online at https://www.mdpi.com/article/10.3390/cancers13112641/s1, Figure S1: RNA-Seq heatmap for top 50 downregulated genes in 4T1.2 vs. 4T1 cells. Relative fold change (regularized-log2) upward genes (red) and downward genes (blue), Figure S1. (cont’d): Representative MRI images of 4T1.2-Luc+ mice (B, D, and F; Day 17, N = 3, coronal projection) and corresponding axial projection images (C, E, and G) were shown. (B–C) Large subcutaneous tumors are located at the resection site. Tumor metastases were observed near the kidneys and in the lungs, Figure S2. MMP13 is downregulated at the metastatic sites vs. primary tumor. mRNA levels were determined by qPCR analysis in the primary tumors, meta-static lesions (A), and corresponding control tissues (B) of mice, as mentioned in Figure 3A. MMP3 mRNA levels were normalized with GAPDH. Data are mean ± SEM, oneway ANOVA P = 0.0001, Tukey’s posthoc test, P < 0.05, n = 3. (C), Table S1. Gene expression by subtypes, stages (AJCC), and Nottingham grades., Table S2. Subtype-specific survival by gene expression (comparing subgroup-specific top and bottom tertiles).
