# Supplementary material for: Altered Expression of Secreted Mediator Genes That Mediate Aggressive Breast Cancer Metastasis to Distant Organs
Source: Cancers (Basel). 2021 May 27;13(11):2641. doi: 10.3390/cancers13112641 (PMC8199412; doi:10.3390/cancers13112641)
Supplement: Supplementary file 1 [file cancers-13-02641-s001.zip › cancers-1207134-supplementary.pdf]

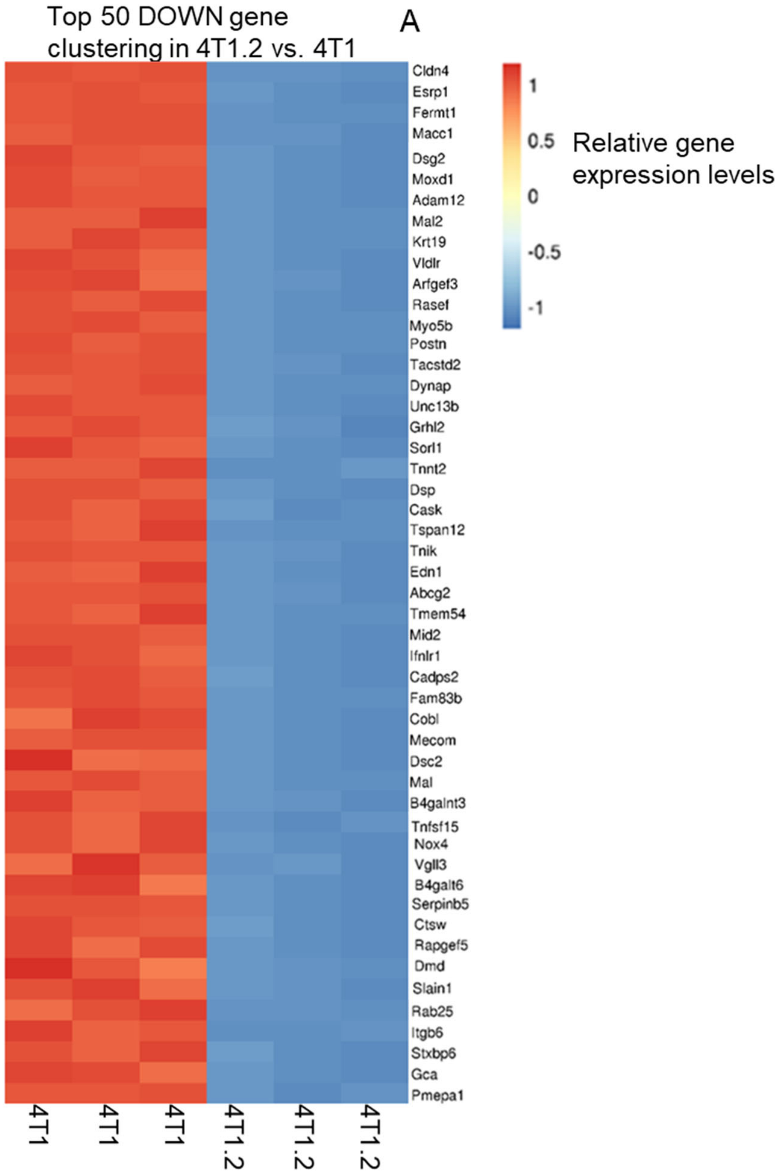

Figure S1. RNA-Seq heatmap for top 50 downregulated genes in 4T1.2 vs. 4T1 cells. Relative fold change (regularized-log2) upward genes (red) and downward genes (blue).

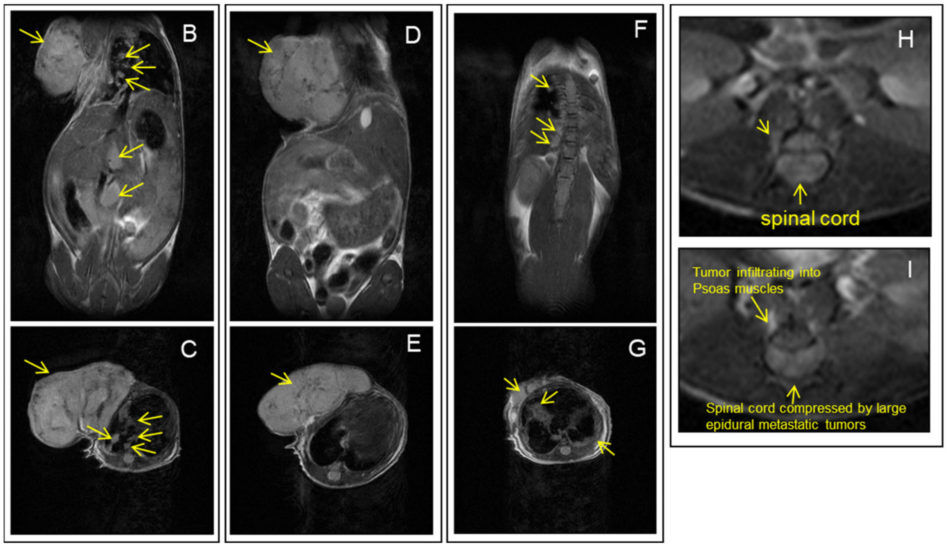

Figure S1. (cont'd): Representative MRI images of 4T1.2-Luc+ mice (**B, D**, and **F**; Day 17, N = 3, coronal projection) and corresponding axial projection images (**C, E**, and **G**) were shown. (**B–C**) Large subcutaneous tumors are located at the resection site. Tumor metastases were observed near the kidneys and in the lungs. Of note, the spleen became enlarged in this mouse. No tumor metastases in the spine or liver were observed in this mouse. Yellow arrows indicate local tumor recurrence and metastasis. (**D–E**) Large subcutaneous tumors are located on the right upper torso, indicating local recurrence. No tumor metastases were observed in the spine or liver of the mouse. (**F–G**) Tumor found in lungs, superior liver/diaphragm boundary, ventral side of the liver, right side of the spine (but seems more associated with liver), and subcutaneous at the resection site and toward the left side of the liver. (**H–I**) More transverse slice images (MRI) are shown from Figure 1G, and data suggested that the mouse has metastatic epidural tumors compressing the spinal cord and tumor infiltration into the psoas muscles.

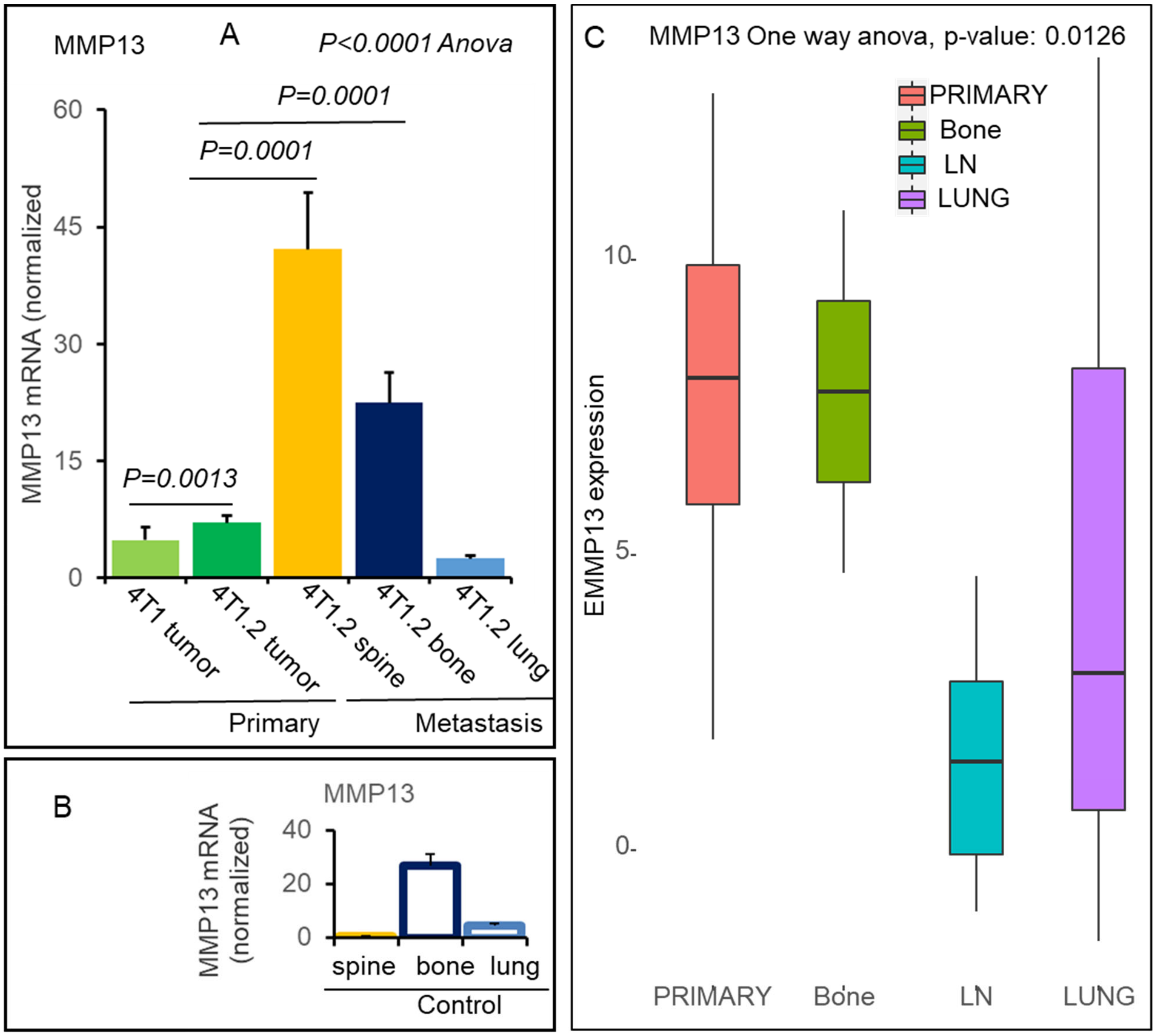

Figure S2. MMP13 is downregulated at the metastatic sites vs. primary tumor. mRNA levels were determined by qPCR analysis in the primary tumors, meta-static lesions (**A**), and corresponding control tissues (**B**) of mice, as mentioned in Fig. 3A. MMP3 mRNA levels were normalized with GAPDH. Data are mean ± SEM, oneway ANOVA P = 0.0001, Tukey's posthoc test, $P < 0.05$, $n = 3$. (**C**) Boxplots show a high MMP13 expression score of primary tumors vs. metastasis to bone, LN, or the lung. Boxplots analyses, Kruskal-Wallis test (non-parametric method), $P < 0.05$; Tukey's t-test with medians and inter-quartile ranges, $P < 0.05$, as a significant difference.

Table S1. Gene expression by subtypes, stages (AJCC), and Nottingham grades.

(A) Gene expression by subtype.

Cohort	Gene	Group size TNBC	HER2+	HER2-HR+	Group mean (log2) TNBC	HER2+	HER2-HR+	Raw Welch t test P TNBC vs. HER2+	TNBC vs. HER2-HR+	HER2+ vs. HER2-HR+
METABRIC	LCN2	299	236	1369	8.469522	7.780776	6.544277	0.00009413231	9.152331E-41	1.296752E-18
METABRIC	S100A8	299	236	1369	9.990452	10.40698	7.564581	0.04266053	3.359314E-56	3.294607E-43
METABRIC	ESM1	299	236	1369	6.649347	6.719099	6.595036	0.2574883	0.2565001	0.008406682
SCANB	LCN2	143	338	2346	4.098149	2.187423	1.128988	0.00000000000145872	1.132423E-28	0.0000000002847784
SCANB	S100A8	143	338	2346	5.548075	5.023384	2.807355	0.04364269	1.811319E-27	4.151621E-33
SCANB	ESM1	143	338	2346	1.762783	1.602724	1.371953	0.1783863	0.0002419187	0.0005219668
TCGA-BRCA	LCN2	160	185	602	4.08845	2.059449	1.010153	0.0000000008815633	4.111218E-25	0.00002655829
TCGA-BRCA	S100A8	160	185	602	4.498863	3.129584	1.30394	0.00005897973	2.026157E-26	0.000000000007487894
TCGA-BRCA	ESM1	160	185	602	2.945419	3.375676	2.934407	0.001127234	0.9175058	0.00004793637

(B) Gene expression by path. stage (AJCC).

Cohort	Gene	Group size I	II	III/IV	Group mean (log2) I	II	III/IV	Raw Welch t test P I vs. II	I vs. III/IV	II vs. III/IV
METABRIC	LCN2	475	800	124	6.759933	7.07102	7.258344	0.0005015975	0.006153155	0.2903485
METABRIC	S100A8	475	800	124	8.105319	8.39943	8.699253	0.01699559	0.01280426	0.1909408
METABRIC	ESM1	475	800	124	6.633889	6.636339	6.648311	0.9532341	0.8504663	0.8701496
TCGA-BRCA	LCN2	180	619	267	1.480439	1.784743	1.66527	0.21436	0.4993634	0.5923742
TCGA-BRCA	S100A8	180	619	267	1.653806	2.286016	2.167981	0.009688262	0.0612003	0.5804114
TCGA-BRCA	ESM1	180	619	267	3.043189	3.098926	2.839195	0.5767635	0.07840467	0.006360458

(C) Gene expression by Nottingham grade.

Cohort	Gene	Group size 1	2	3	Group mean (log2) 1	2	3	Raw Welch t test P 1 vs. 2	1 vs. 3	2 vs. 3
METABRIC	LCN2	165	740	928	6.478976	6.679453	7.375742	0.03460408	3.249633E-17	6.933247E-18
METABRIC	S100A8	165	740	928	7.188832	7.658291	9.073655	0.00002084037	1.903476E-47	3.817944E-41
METABRIC	ESM1	165	740	928	6.516959	6.587027	6.662715	0.2452157	0.01494374	0.02528794
SCANB	LCN2	483	1509	1161	1.083967	1.262445	2.08751	0.06625012	3.850869E-17	4.611892E-16
SCANB	S100A8	483	1509	1161	2.455612	2.794114	4.44778	0.00005978779	1.026717E-71	1.476418E-65
SCANB	ESM1	483	1509	1161	1.16149	1.373742	1.67088	0.0002581912	7.884401E-17	0.00000000006352132

Table S2. Subtype-specific survival by gene expression (comparing subgroup-specific top and bottom tertiles).

Cohort	Subtype	Gene	Outcome	Logrank test P	HR	Lower 95% CI of HR	Upper 95% CI of HR
SCANB	TNBC	LCN2	OS	0.8311965	1.083798	0.5163735	2.274745
SCANB	TNBC	S100A8	OS	0.7127072	0.8543891	0.3704025	1.970777
SCANB	TNBC	ESM1	OS	0.4688024	1.321067	0.6211149	2.809815
SCANB	HER2+	LCN2	OS	0.2868995	0.6643575	0.3167014	1.39365
SCANB	HER2+	S100A8	OS	0.600401	0.8101639	0.3698727	1.774571
SCANB	HER2+	ESM1	OS	0.0171431	2.245766	1.188923	4.242043
SCANB	HER2-HR+	LCN2	OS	0.2498725	0.8544435	0.6539954	1.116329
SCANB	HER2-HR+	S100A8	OS	0.0007496382	1.609074	1.223494	2.116169
SCANB	HER2-HR+	ESM1	OS	0.2057267	1.190957	0.9086451	1.560983
TCGA-BRCA	TNBC	LCN2	OS	0.6863739	0.8201269	0.3077023	2.185905
TCGA-BRCA	TNBC	LCN2	DSS	0.7143508	0.7910776	0.227982	2.74497
TCGA-BRCA	TNBC	LCN2	PFI	0.4281276	0.6726905	0.2586134	1.749765
TCGA-BRCA	TNBC	S100A8	OS	0.9031028	1.062258	0.3969068	2.842963
TCGA-BRCA	TNBC	S100A8	DSS	0.7640907	1.22241	0.3309129	4.515645
TCGA-BRCA	TNBC	S100A8	PFI	0.8830963	0.9330898	0.3701882	2.351929
TCGA-BRCA	TNBC	ESM1	OS	0.5326945	1.329982	0.5391484	3.280826
TCGA-BRCA	TNBC	ESM1	DSS	0.3297474	1.753202	0.5614073	5.47502
TCGA-BRCA	TNBC	ESM1	PFI	0.690275	1.205813	0.476718	3.049989
TCGA-BRCA	HER2+	LCN2	OS	0.8404712	1.133668	0.3381042	3.801205
TCGA-BRCA	HER2+	LCN2	DSS	0.3659778	2.627268	0.4378135	15.76593
TCGA-BRCA	HER2+	LCN2	PFI	0.403331	1.760757	0.5035109	6.157293
TCGA-BRCA	HER2+	S100A8	OS	0.9077254	0.939664	0.3243433	2.722327
TCGA-BRCA	HER2+	S100A8	DSS	0.6267605	1.515888	0.301287	7.627002
TCGA-BRCA	HER2+	S100A8	PFI	0.3266565	2.173739	0.5339387	8.849595
TCGA-BRCA	HER2+	ESM1	OS	0.2602478	1.738325	0.6522829	4.632614
TCGA-BRCA	HER2+	ESM1	DSS	0.03435578	6.291787	1.570462	25.20697
TCGA-BRCA	HER2+	ESM1	PFI	0.02048559	4.075747	1.366649	12.15506
TCGA-BRCA	HER2-HR+	LCN2	OS	0.02004697	0.4911698	0.266111	0.9065684
TCGA-BRCA	HER2-HR+	LCN2	DSS	0.06990621	0.4529299	0.1906185	1.07621
TCGA-BRCA	HER2-HR+	LCN2	PFI	0.06208435	0.5568002	0.2977292	1.041303
TCGA-BRCA	HER2-HR+	S100A8	OS	0.1771771	0.6283462	0.3204893	1.231925
TCGA-BRCA	HER2-HR+	S100A8	DSS	0.7532274	0.8692657	0.3610349	2.092936
TCGA-BRCA	HER2-HR+	S100A8	PFI	0.4775851	0.7968914	0.4252217	1.493423
TCGA-BRCA	HER2-HR+	ESM1	OS	0.4214821	1.284964	0.6960809	2.37204
TCGA-BRCA	HER2-HR+	ESM1	DSS	0.2860177	1.617747	0.6729301	3.889119
TCGA-BRCA	HER2-HR+	ESM1	PFI	0.304155	1.384029	0.7437917	2.575366
METABRIC	TNBC	LCN2	OS	0.1690644	0.7712996	0.5325019	1.117185
METABRIC	TNBC	LCN2	DSS	0.6270566	0.8971671	0.5788116	1.390623
METABRIC	TNBC	LCN2	RFS	0.92731	0.9794575	0.6265823	1.531063
METABRIC	TNBC	S100A8	OS	0.8797066	1.028408	0.7146087	1.480004
METABRIC	TNBC	S100A8	DSS	0.4760827	0.8558579	0.558021	1.312661
METABRIC	TNBC	S100A8	RFS	0.7214892	0.9233252	0.5956764	1.431195
METABRIC	TNBC	ESM1	OS	0.06785994	1.405336	0.9650234	2.046551
METABRIC	TNBC	ESM1	DSS	0.02713191	1.671391	1.05759	2.641429
METABRIC	TNBC	ESM1	RFS	0.06816303	1.531018	0.9659142	2.426733
METABRIC	HER2+	LCN2	OS	0.2602412	1.259213	0.841578	1.8841
METABRIC	HER2+	LCN2	DSS	0.2243841	1.32091	0.8418642	2.072547
METABRIC	HER2+	LCN2	RFS	0.6255719	1.119086	0.7113869	1.76044
METABRIC	HER2+	S100A8	OS	0.1310709	1.351065	0.9093351	2.007376

METABRIC	HER2+	S100A8	DSS	0.08080924	1.496141	0.9500641	2.356092
METABRIC	HER2+	S100A8	RFS	0.1396694	1.416424	0.8916025	2.25017
METABRIC	HER2+	ESM1	OS	0.2023138	1.304144	0.8662699	1.963351
METABRIC	HER2+	ESM1	DSS	0.4882853	1.170697	0.7486584	1.830649
METABRIC	HER2+	ESM1	RFS	0.4749246	1.177973	0.7512301	1.847131
METABRIC	HER2-HR+	LCN2	OS	0.4746962	0.9404932	0.7947507	1.112962
METABRIC	HER2-HR+	LCN2	DSS	0.5839843	1.068142	0.8436372	1.352392
METABRIC	HER2-HR+	LCN2	RFS	0.2090304	1.151971	0.9239982	1.436189
METABRIC	HER2-HR+	S100A8	OS	0.2791226	1.098897	0.9256352	1.30459
METABRIC	HER2-HR+	S100A8	DSS	0.02653029	1.316222	1.032436	1.678012
METABRIC	HER2-HR+	S100A8	RFS	0.1268167	1.190772	0.9516329	1.490005
METABRIC	HER2-HR+	ESM1	OS	0.008429306	1.255485	1.058645	1.488925
METABRIC	HER2-HR+	ESM1	DSS	0.006059468	1.392947	1.098825	1.765796
METABRIC	HER2-HR+	ESM1	RFS	0.007516095	1.357008	1.084659	1.697742
